# Effects of integrated care on health outcomes in patients with diabetes in primary healthcare: A systematic review and meta-analysis protocol^☆^

**DOI:** 10.1016/j.mex.2025.103628

**Published:** 2025-09-15

**Authors:** Woska Pires da Costa, Heloísa de Carvalho Torres, Thiago Dias Sarti, Ana Paula Santana Coelho Almeida, Sandro Rogério Rodrigues Batista, Luiz Carlos de Abreu

**Affiliations:** a Instituto Federal Goiano - Campus Morrinhos, Research Department, BR-153 Highway, Km 633, P.O. Box 92, Rural Area, 75658-899, Morrinhos, GO, Brazil; b Universidade Federal do Espírito Santo (UFES), Department of Integrated Health Education, Marechal Campos Avenue, No 1468, Maruípe, 29040-090, Vitória, ES, Brazil; c Universidade Federal de Goiás (UFG), Department of Internal Medicine, 5th Avenue, Unnumbered, East University Sector, 74605-040, Goiânia, GO, Brazil

**Keywords:** Diabetes mellitus, Glycemic control, Healthcare policy, Clinical outcomes, Evidence-based practices, Systematic review, Meta-analysis

## Abstract

Diabetes is a leading global cause of morbidity and mortality among non-communicable diseases, with increasing prevalence and a disproportionate impact on low- and middle-income countries. Integrated care has emerged as a promising approach for addressing the challenges of diabetes management, thereby enhancing clinical outcomes and optimizing healthcare resource allocation. This protocol for a systematic review and meta-analysis aims to evaluate the effects of integrated care on clinical and health outcomes in individuals with diabetes who are followed up in primary care (PC):

● The method follows PRISMA-P 2015, PRISMA-S, and PRESS 2015 guidelines.

● A comprehensive search will be conducted in Scopus, Web of Science, PubMed, Embase, and CINAHL, supplemented by evidence mapping using Litmaps.

● Risk of bias will be assessed with validated tools, and the overall certainty of the evidence evaluated using GRADE.

Eligible studies will include those evaluating the effects of integrated care on clinical and health outcomes in individuals with diabetes treated in PC. The studies will be screened, and data will be synthesized through a meta-analysis. The findings may contribute to evidence-based public policies and the achievement of SDG-3. By encouraging the adoption of patient-centered approaches, strengthening interprofessional collaboration, and training aspects.

## Graphical abstract

**Figure:**
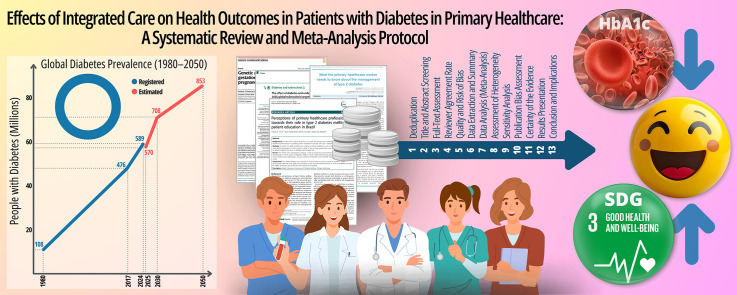

## Specifications table

**Table:** 

**Subject area**	Medicine and Dentistry
**More specific subject area**	Primary Health Care
**Name of your method**	Systematic Review and Meta-Analysis
**Name and reference of original method**	Preferred Reporting Items for Systematic Review and Meta-Analysis Protocols (PRISMA-P 2015)
**Resource availability**	Not applicable – this is a secondary data study based on published literature.

## Background

Diabetes mellitus is one of the most prevalent chronic non-communicable diseases (NCDs) worldwide, posing a major public health challenge, particularly in low- and middle-income countries (LMICs) [1]. In 2024, an estimated 589 million adults aged 20 to 79 were living with diabetes globally, with projections suggesting this number will rise to 853 million by 2050. The disease was responsible for >3.4 million deaths in 2024 alone, and direct healthcare costs surpassed USD 1 trillion for the first time [2]. Population aging and increasing early-onset diabetes have led to substantial impacts on healthcare systems, social productivity, and quality of life [1].

Diabetes is closely associated with modifiable risk factors such as obesity, unhealthy diets, physical inactivity, and other lifestyle-related conditions [1,[3], [4], [5]]. These factors contribute to serious complications – including cardiovascular, renal, and neurological diseases – which further elevate the risk of premature mortality and disability [1,4,[6], [7], [8]]. Between 1980 and 2017, the global diabetic population increased from approximately 108 million to 476 million, with estimates suggesting >570 million by 2025 and nearly 7079 cases per 100,000 people by 2030 [1,4,8]. This upward trend is particularly pronounced in regions such as Central Asia, South Asia, and Sub-Saharan Africa [4,9,10]. These regional disparities underscore the pressing need for targeted interventions addressing the unique challenges faced by vulnerable populations.

To address the increasing burden of NCDs, the World Health Organization (WHO) launched the Global Action Plan for the Prevention and Control of NCDs 2013–2020, targeting a 25 % reduction in premature mortality [11]. However, projections indicate that, without effective interventions, the global prevalence of diabetes could reach 18 % by 2030, compromising progress toward Sustainable Development Goal (SDG) 3.4 [[11], [12], [13]]. This underscores the importance of prioritizing diabetes prevention and management in national and international health agendas.

In this context, integrated care – also referred to as coordinated, comprehensive, or team-based care – has emerged as a promising strategy for managing complex chronic diseases such as diabetes. Grounded in the Chronic Care Model (CCM), integrated care supports clinical decision-making, patient self-management, and optimization of information systems [14]. This model focuses on improving health outcomes, enhancing the patient experience, and optimizing resource use through coordination among healthcare providers and across different levels of care [11,14,15].

Evidence suggests that CCM-based interventions can lead to improved glycemic control, fewer hospitalizations, and enhanced quality of life among individuals receiving diabetes care in primary healthcare settings [16]. Nevertheless, systematic reviews in this field often encompass a broad range of NCDs or are limited to high-income countries, reducing the external validity of findings for LMIC contexts [17]. Furthermore, the variability in how integrated care is defined and implemented across health systems presents methodological challenges for comparing results and synthesizing conclusions [11]. These gaps necessitate rigorous reviews that assess the impact of integrated care models on diabetes outcomes. Thus, this study protocol outlines the method for a systematic review to evaluate the effects of integrated care on health and clinical outcomes in patients with diabetes treated in primary healthcare services.

## Method details

This systematic review protocol is designed to synthesize the existing scientific evidence on the effects of integrated care in primary healthcare services on clinical and health outcomes in patients with diabetes. The methodology follows internationally recognized guidelines for conducting systematic reviews, ensuring transparency, rigor, and reproducibility. All procedures – from formulating the research question to data analysis – were predefined and were thoroughly detailed in this protocol. This approach enables the identification of any methodological deviations and ensures that the review adheres to the highest standards of scientific integrity.

### Context and rationale for method choice

In addition to the concerning global indicators presented in the Background section, patient-centered communication – marked by respectful dialogue, active listening, and sensitivity to patients' values and preferences – is fundamental to effective diabetes care. It contributes to better treatment adherence, glycemic control, and overall quality of life [18]. Managing diabetes also requires interdisciplinary teamwork involving physicians, nurses, nutritionists, physical educators, pharmacists, dentists, podiatrists, and mental health professionals. Equally important is the active role of the patient in co-developing self-care plans and implementing behavioral changes in partnership with their healthcare team. Such collaborative care models have the potential to transform chronic disease management by fostering shared responsibility and empowerment.

Despite growing interest in integrated care, a shortage of systematic reviews focusing specifically on its effects for patients with diabetes in primary care (PC) settings remains. Investigating these effects is essential for informing evidence-based public health strategies, strengthening health systems, and supporting countries in achieving global health targets. As the prevalence of diabetes continues to rise, prevention, early diagnosis, and effective clinical management must remain priorities in the formulation of public health policies aimed at controlling this global epidemic. A comprehensive understanding of how integrated care influences clinical outcomes, patient experience, and system efficiency could offer invaluable insights for designing more effective, equitable, and sustainable approaches to diabetes care worldwide.

Therefore, systematic review and meta-analysis are the most appropriate methods to synthesize the current evidence on integrated care interventions for diabetes management in PC settings. This approach enables a comprehensive understanding of how integrated care impacts clinical outcomes, patient experience, and system efficiency, providing invaluable insights for designing more effective, equitable, and sustainable approaches to diabetes care globally. Moreover, it enables the identification of gaps in current research, the assessment of methodological quality and heterogeneity between studies, and the establishment of best practices for conducting future investigations. By identifying improvements in key outcomes, the findings may contribute to the development of public health policy, support evidence-based decision-making, and guide the refinement of integrated care strategies, as well as the efficient allocation of resources for managing chronic conditions at the population level.

### Protocol, registration, and ethical issues

This systematic review protocol was developed according to established methodological standards for systematic reviews, and its reporting follows the Preferred Reporting Items for Systematic Reviews and Meta-Analyses for Protocols 2015 (PRISMA-P 2015) [19,20], as detailed in the supplementary material (S1 File). It has been registered in PROSPERO (Register No. CRD420251107477, July 21, 2025), as it addresses a research question directly related to human health [21]. In addition to this registration, this study protocol is being published in this scientific journal before data extraction and the start of the review, aiming to ensure methodological rigor and promote research transparency [22]. Prospective registration plays a crucial role in disclosing the study's methodological details, enabling peers to verify whether all procedures were conducted and reported as initially planned [17]. Any amendments to the protocol during the study will be updated in the registration record and clearly reported in the final manuscript presenting the systematic review results [23]. Furthermore, this type of study does not require ethical approval, as it involves no direct interaction with human participants and relies solely on secondary data from previously published sources [24].

### Primary search strategy and databases

This systematic review will identify and gather scientific evidence from multidisciplinary databases – Scopus™ and Web of Science™ Core Collection – and specific health databases – MEDLINE/PubMed® via the National Library of Medicine® interface, Embase™, and Cumulative Index to Nursing and Allied Health Literature (CINAHL®) via EBSCOhost. For systematic searches, a pre-established search string will be employed to identify studies aligned with the focus of this systematic review [23]. Furthermore, the research team will adapt the search strategy for each selected database, incorporating all identified keywords and indexing terms [22]. These databases were selected based on identifying relevant studies from prior research to meet the study's objective.

The search strategy for this protocol was developed based on the main concepts of the research question and the study's inclusion criteria. To enhance accuracy, the syntax will be adapted to meet the specific requirements of each database, utilizing advanced search features [23]. The searches were performed within the metadata fields of titles, abstracts, and keywords in the selected scientific databases [25]. The eligibility criteria were determined using the Population, Intervention, Comparison, Outcome, and Study design (PICOS) [26], a framework for evidence-based health research. The intervention block was divided into two distinct components: one representing the care model and the other representing the support level, allowing for a more focused and structured search. Accordingly, the following parameters were established: *P* = patients adults with diabetes receiving primary healthcare; *I* = integrated care (care model) implemented within primary healthcare services (support level); C = patients with diabetes who do not use this healthcare services (this component is often implicit in the studies and, therefore, does not need to be explicitly included in the search strategy, as doing so may unnecessarily restrict the retrieval of relevant records; thus, the comparison component will be identified and analyzed at the data screening stage); O = clinical and health outcomes (e.g., glycemic control, treatment adherence, quality of life, hospitalization, among others); and S = randomized controlled trial, clinical trial, quasi-experimental, non-randomized, intervention, observational, cohort, longitudinal and comparative, all of which are health study designs that allow for the comparison of outcomes between groups.

The search string was developed by incorporating synonyms, related terms, variations between British and American English, singular and plural forms, and other relevant linguistic nuances [23]. To maximize the retrieval of pertinent studies, indexed terms were combined with their synonyms or equivalent terms using the Boolean operator "OR", and these groups were subsequently linked with the "AND" operator [25]. The search will be performed using the terms specified in the strings (Table 1).

**Table 1:** Keywords included in the search strategy are organized in blocks.

Blocks (PICOS)	Keywords used
**#1** P	(diabetes OR diabetic OR "t2dm" OR "glucose intolerance" OR "impaired glucose tolerance" OR glycuresis OR hyperglycemia OR "high blood sugar" OR glucosuria)
**#2** I (care model)	("integrated care" OR "integrated healthcare" OR "integrated health-care" OR "integrated health care" OR "coordinated care" OR "coordinated healthcare" OR "coordinated health-care" OR "coordinated health care" OR "multidisciplinary care" OR "multidisciplinary healthcare" OR "multidisciplinary health-care" OR "multidisciplinary health care" OR "comprehensive care" OR "comprehensive healthcare" OR "comprehensive health-care" OR "comprehensive health care" OR "collaborative care" OR "collaborative healthcare" OR "collaborative health-care" OR "collaborative health care" OR "interprofessional care" OR "interprofessional healthcare" OR "interprofessional health-care" OR "interprofessional health care" OR "team-based care" OR "team-based healthcare" OR "team-based health-care" OR "team-based health care" OR "continuity of care" OR "continuity of healthcare" OR "continuity of health-care" OR "continuity of health care" OR "patient-centered care" OR "patient-centered healthcare" OR "patient-centered health-care" OR "patient-centered health care" OR "chronic care model" OR "chronic healthcare model" OR "chronic health-care model" OR "chronic health care model")
**#3** I (support level)	("primary medical care" OR "primary care" OR "primary healthcare" OR "primary health care" OR "primary health-care" OR "family healthcare" OR "family health care" OR "family health-care" OR "family health program" OR "family healthcare program" OR "family health care program" OR "family health-care program" OR "community health services" OR "community healthcare services" OR "community health-care services" OR "general practice" OR "general healthcare practice" OR "general health care practice" OR "general health-care practice" OR "general practitioner" OR "general healthcare practitioner" OR "general health care practitioner" OR "general health-care practitioner" OR "first-level care" OR "first-level healthcare" OR "first-level health care" OR "first-level health-care" OR "ambulatory care" OR "ambulatory healthcare" OR "ambulatory health-care" OR "ambulatory health care" OR "outpatient care" OR "outpatient healthcare" OR "outpatient health-care" OR "outpatient health care")
**#4** O	("clinical outcome" OR "health outcome" OR "glycemic control" OR "blood glucose" OR "HbA1c" OR "hemoglobin A1c" OR "treatment adherence" OR "medication adherence" OR "self-care" OR "disease management" OR "life prospect" OR "life expectation" OR "perspective on life" OR "prospect in life" OR "expectation of life" OR "expectation for life" OR "diabetes control" OR "metabolic control" OR "complication rate" OR "diabetes complications" OR "functional status" OR "treatment outcome" OR "quality of life" OR qol OR hospitalization OR mortality OR readmission)
**Search string:**	**(#1) AND (#2) AND (#3) AND (#4)**

**Note:** PICOS stands for Population, Intervention, Comparison, Outcome, Study design; furthermore, it is a framework created to define the issue addressed by the systematic review. The block C of this framework is implicit in the studies, which is why it was not included in the search strategy, and block S will be identified using the software adopted to manage the systematic review.

The search strategy was validated using the evidence-based checklist provided by the Peer Review of Electronic Search Strategies (PRESS 2015) guideline [27]. The syntax was then tailored to the specifications of each database to expand the search scope and ensure the inclusion of relevant studies. The searches across all databases included in this systematic review will be performed in a single day, and the corresponding metadata files will be obtained directly from the database's respective platforms based on the systematic search string [23]. The entire extraction process will be recorded by the PRISMA for Search (PRISMA-S) guidelines [28] (see S2 File).

### Secondary searches

**Fig. 1:**
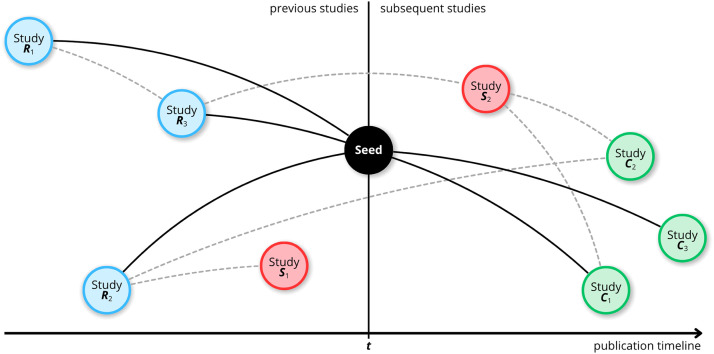
Secondary search process based on seed articles to generate the citation map.

**Note:** The black circle serves as the seed study and was selected as the pivot for mapping the evidence; the blue circles – prior studies (*R*_1_, *R*_2_, and *R*_3_) directly cited by the seed; the green circles – more recent studies (*C*_1_, *C*_2_, and *C*_3_) that cite the seed; and the red circle – similar to the seed, identified as relevant by an artificial intelligence algorithm based on the seed (*S*_1_ and *S*_2_, linked by dashed lines).

Secondary searches will be conducted through citation tracking to identify additional relevant literature [29] by mapping citations from articles included in the initial phase of the systematic review [30], as well as from review articles that, although excluded, were flagged for this purpose due to their partial relevance to the research topic [31]. This process will be carried out using the Litmaps® platform (https://www.litmaps.com/), which employs artificial intelligence to locate related studies based on a reference article (called seed) and maps scientific evidence through cross-references and citations [31] (Fig. 1). For each included article, a search will be performed in Litmaps® using its respective digital object identifier (DOI) [30].

### Eligibility criteria

This systematic review will include relevant articles without language restrictions or publication dates that meet the inclusion criteria [23]. Some eligible articles may be excluded if they meet at least one of the predefined exclusion criteria [25,32,33]. The eligibility criteria were mainly defined using the PICOS framework, as detailed below.

**Inclusion criteria:**

**(i1)** Evidence from studies retrieved from scientific databases using the strategy previously defined with a primary systematic search string [23] or by secondary searches.
**(i2)** Peer-reviewed journal articles published as original studies [32,33].
**(i3)** Population: Adults with diabetes receiving primary healthcare.
**(i4)** Intervention: Integrated care models implemented in primary healthcare services.
**(i5)** Outcomes: Clinical and health outcomes (e.g., glycemic control, treatment adherence, quality of life, hospitalization, mortality) in studies evaluated the effects of integrated care in primary healthcare services on patients with diabetes.
**(i6)** Study design: Eligible study designs will include comparative interventional studies (such as randomized controlled trials and quasi- or non-randomized clinical trials), and comparative observational studies (such as cohort or other longitudinal designs). Review studies will be identified and labeled for citation mapping; however, the results of these reviews will not be included in the meta-analysis, as they are more susceptible to interpretation bias.

**Exclusion criteria:**

**(e1)** Duplicates: If multiple articles have been published by the same author, on the same dataset, and on the same topic, only the most comprehensive among them will be considered. Duplicates will be removed following Bramer's method [34], and a manual review will be conducted to confirm their exclusion [32,35].
**(e2)** Opinion articles, commentaries, editorials (or similar), dissertations, theses, reviews, animal studies, articles in press, journal letters, and books [36].
**(e3)** Studies that are not fully available in the databases searched and cannot be accessed even after attempts to contact the authors have been made [32,33,37].
**(e4)** Articles written in a restricted language that cannot be appropriately translated [38]. This criterion will apply only after exhausting all translation options – including international collaborators, artificial intelligence tools, and specialized services – and will be reported in the review findings [25].
**(e5)** Studies with retraction records [38].

Regarding the last criterion described, considering the substantial variability in how retractions are disseminated, which may result in retracted articles still being cited and included in studies, it is necessary to perform retraction checks [39]. The validity of eligible studies will be assessed, and any retraction records will be identified using the Scite tool, an acronym for "*Smart citation index*" (available at https://scite.ai/) [40]. This tool offers several functionalities, including the ability to determine whether a given article has been retracted or has received critical citations [41].

### Risk of bias assessment

The risk of bias will be assessed according to the study design and focused on the outcomes of interest for this review (e.g., glycemic control, treatment adherence, hospitalizations, and quality of life). Randomized controlled trials will be evaluated with the Risk of Bias 2.0 (RoB 2.0) tool [42], applied to specific study outcomes. Non-randomized studies will be assessed with the "*Risk Of Bias In Non-randomized Studies of Interventions*" (ROBINS-I) [43], which also evaluates bias at the outcome level. Cohort studies will be appraised using the Newcastle-Ottawa Scale (NOS) [44,45].

Two reviewers will independently apply the tools, with disagreements resolved by discussion or by a third reviewer. The results of the risk of bias assessments will be presented in summary tables and figures, and will be considered in the interpretation of findings and in sensitivity analyses.

### Review process

**Fig. 2:**
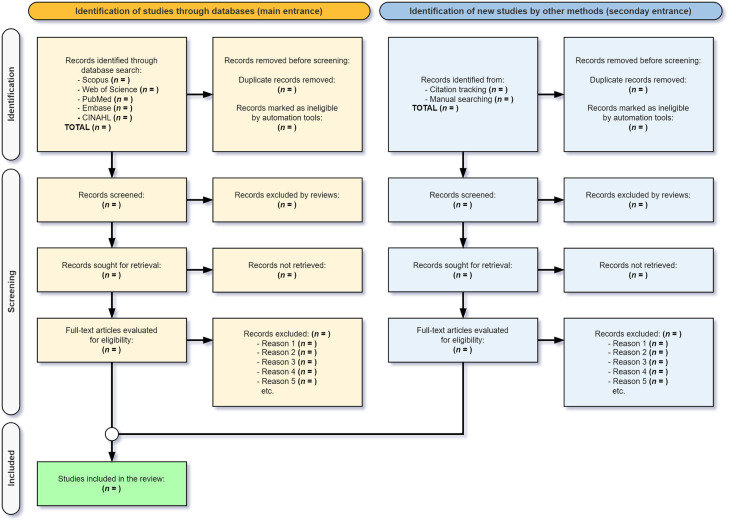
PRISMA 2020 flow diagram for identifying, screening, and including studies in the review.

The screening process described in this protocol outlines a priori methodological steps for conducting this systematic review and meta-analysis, as follows:

1. Metadata retrieved from the selected databases will be imported into Rayyan® software (Rayyan Systems Inc., Cambridge, MA, USA), a web-based tool designed to facilitate systematic review screening [46].
2. A deduplication process will be performed by one reviewer (R1) to remove duplicate records [30].
3. Filters will be applied in Rayyan® to exclude study types that do not meet the inclusion criteria, thereby enhancing the specificity and sensitivity of the search. This pre-filtering step will be conducted before the screening phase, with reviewers appropriately labeling the materials, to optimize the screening process and the review timeline.
4. Title and abstract screening will be conducted independently by two reviewers (R1 and R2), with blind mode using Rayyan®; studies will be assessed based on predefined eligibility criteria, with each record labeled as "include" or "exclude".
5. The agreement rate between reviewers will be calculated [38], along with Cohen's kappa coefficient – denoted by κ [47,48] –, to measure inter-rater reliability [25].
6. Disagreements between reviewers will be resolved by a third senior reviewer (R3) based on previously established eligibility criteria [25,49].
7. Two reviewers (R1 and R2) will independently retrieve and assess the full texts of the studies selected in the previous stage to confirm their eligibility for final inclusion, as well as to perform a new labeling process. Any disagreements between them will be resolved by a third senior reviewer (R3). When a single study has multiple publications, all related reports will be grouped and considered together to ensure that relevant data from different papers are accurately captured without duplication.
8. The quality and risk of bias of the included studies will be assessed using validated tools and standardized procedures (RoB 2.0, ROBINS-I, and Newcastle-Ottawa) to ensure consistent and transparent appraisal supervised by a senior reviewer (R3) and to guide the interpretation of findings and support planned sensitivity analyses.
9. The risk of bias in the included studies will be assessed using validated tools appropriate to each study design, and the overall certainty of the evidence will be appraised using the GRADE approach.
10. A summary table of the studies included will be constructed.
11. The studies will be analyzed, and quantitative data will be extracted for meta-analysis in accordance with the Cochrane Handbook for Systematic Reviews of Interventions [50] (further details are provided below).
12. The findings will be reported, and conclusions will be drawn from the synthesized evidence.
  All decisions made for each study will be duly labeled in Rayyan® to ensure precise, transparent, and complete documentation of the entire process, including both inclusion and exclusion, whether at the first or second stage of the review. Lastly, the reporting of this systematic review will adhere to the PRISMA 2020 guidelines, as outlined in the study selection process flowchart [51] (Fig. 2).

### Review training

A pilot assessment will be conducted to train the reviewers involved in this study under the supervision of an experienced researcher [23]. The objectives of this training session are to standardize screening decisions based on the pre-established eligibility criteria for this systematic review [31]. It will also include guidance on using the Rayyan® software, ensuring reviewers become familiar with its features and functionalities, as well as promoting a standardized analysis process and helping reviewers correctly label the studies at each review stage [23]. Training will also include orientation on risk-of-bias tools according to study design (RoB 2.0, ROBINS-I, and NOS), as well as instruction on conducting sensitivity and meta-regression analyses using both frequentist frameworks.

### Data extraction

All data extracted from the evidence sources will be entered into a summarized table that includes information such as the study, source, objective, population, sample size, time frame, method, main results, primary and secondary outcomes, gaps, and other relevant findings (see S3 File). This table will be reviewed and adjusted as necessary during the data extraction process [37,52,53]. If a study is unavailable, one reviewer will contact the corresponding author to request the full text [49] and, if necessary, the related dataset. The full extracted evidence will be compiled as supplementary material and made available alongside the systematic review publication as "Evaluated Data" [23].

### Outcomes of interest

The primary outcome of interest will be glycemic control, assessed by glycated hemoglobin (HbA1c) levels at the latest reported follow-up. For the secondary outcomes, it will be considered:

• Treatment adherence: measured by validated self-report instruments, prescription refill data, or clinical records.
• Hospitalizations: all-cause and diabetes-related, as reported in each study.
• Mortality: all-cause and diabetes-related.
• Diabetes complications: including microvascular and macrovascular events.
• Quality of life: assessed using validated instruments such as the EQ-5D [54,55], the 36-item Short-Form (SF-36) [56,57], the World Health Organization Quality of Life (WHOQOL) [58,59], among others.
• Use of healthcare services: such as emergency visits, primary care consultations, or referrals.

Outcomes will be collected as reported by the study authors. When available, results will be grouped by follow-up periods (short-term: <12 months; medium-term: 12–24 months; long-term: >24 months).

### Data synthesis

For meta-analyses, a minimum of two comparable studies will be required to perform quantitative synthesis. When only a small number of studies are available, pooled estimates will be interpreted with caution, and findings will be complemented by narrative synthesis. When data relevant to outcomes of interest are missing or unclear, study authors will be contacted to obtain additional information. If data remain unavailable, the impact of missing information will be explored through sensitivity analyses, or results will be reported narratively.

### Data analysis

Quantitative synthesis will focus on evaluating the effects of integrated care on patients with diabetes in primary healthcare settings. Data extraction will be structured around key outcomes, and sociodemographic groups will be used to stratify the results, allowing for the identification of patterns and disparities. Meta-analyses will be conducted using Comprehensive Meta-Analysis (CMA) software, version 4.0 for Microsoft Windows™ (Biostat, Inc., Englewood, NJ, USA) [60]. The pooled effect size (*ES*) will be calculated using a random-effects model (95 % CI). To complement this, an exploratory random-effects meta-analysis will also be performed, providing posterior distributions and probabilistic interpretations, which are particularly beneficial in the presence of sparse data or high heterogeneity.

For randomized and non-randomized interventional studies, effect measures such as risk ratios (RR), odds ratios (OR), mean differences (MD), or standardized mean differences (SMD) will be extracted, depending on the type of outcome. For observational studies (e.g., cohort designs), risk ratios (RR), hazard ratios (HR), and odds ratios (OR) will be extracted.

Interventional and observational studies will be analyzed separately in the primary analyses. If sufficient studies are available, subgroup or sensitivity analyses will be conducted to compare results across study designs. Any differences in findings will be interpreted cautiously, considering study design, risk of bias, and methodological quality.

Special consideration will be given to unit of analysis issues. In the case of cluster-randomized controlled trials, effect estimates that account for the clustering will be extracted whenever reported by the study authors. If such adjustments are not provided, we will calculate the adequate sample size using the design effect, based on the intraclass correlation coefficient (ICC). When the ICC is not available, values from similar studies or recommendations from the literature will be used. Sensitivity analyses will be conducted to explore the impact of including cluster trials alongside individually randomized trials.

#### Heterogeneity

Heterogeneity will be assessed using Cochran's *Q* test (with *p* < 0.05 indicating significant heterogeneity) and quantified by the *I*^2^ statistic, interpreted as low (0–25 %), moderate (26–50 %), or high (>50 %) heterogeneity [61]. Predefined clinical thresholds for effect sizes will guide interpretation (e.g., an HbA1c reduction of 0.5 % or greater is considered clinically meaningful). To further explore heterogeneity and assess robustness, the following analyses will be undertaken: (1) Subgroup analyses, based on categorical variables such as age group, gender, and type of integrated care components (e.g., multidisciplinary teams, care coordination, patient education) [30]; (2) meta-regression analyses will be performed to examine the influence of continuous moderators [62] such as age, duration of diabetes, frequency of follow-up visits, number of care components, and baseline glycemic control; (3) influence diagnostics, including leave-one-out analyses and Baujat plots [63], to detect studies exerting undue influence on heterogeneity or overall estimates; and (4) separate meta-analyses will be conducted according to the study design to maintain methodological rigor [30]. *ES* reported in the included studies will be used. When available, adjusted *ES* will be prioritized as they account for potential confounders. If only crude *ES* is reported, these will be included to maximize data utilization [30].

Given the variability in how integrated care is defined across health systems and studies, we will extract and document the operational definition and components of integrated care used in each study (e.g., multidisciplinary teams, care coordination, patient education, or Chronic Care Model-based interventions). To address this heterogeneity, subgroup analyses will be conducted according to the type and intensity of integrated care components, and sensitivity analyses will be used to examine the robustness of the pooled results. Differences in definitions will also be explicitly reported in the results tables and considered in the interpretation of findings.

#### Sensitivity analyses

To assess the robustness of the findings, sensitivity analyses will be conducted by (1) iteratively removing one study at a time (leave-one-out approach) to evaluate its impact on the overall *ES*, and (2) stratifying studies according to their risk of bias levels (low, moderate, high) [62]. These strategies will allow the evaluation of the robustness of the findings and assess whether study quality influences the pooled results.

#### Publication bias

Publication bias will be assessed through both visual and statistical methods. A funnel plot will be generated when at least ten studies are included in the meta-analysis, as recommended by the Cochrane Handbook, due to the low power of visual and statistical tests with smaller samples [50]. For meta-analyses with fewer than ten studies, Begg’s Rank Correlation Test will be applied [64]. When there are ten or more studies, the Egger’s regression test will be used to detect potential asymmetry, with statistical significance set at *p* < 0.05 [65]. To adjust for potential publication bias, (1) the Trim and Fill method will be employed to estimate the number of missing studies and provide corrected *ES* estimates [64,66]; the fail-safe *N* will be calculated to determine the number of missing studies that would be required to nullify the observed effect, thereby assessing the robustness of the findings [64]; and (3) *P*-curve analysis [[67], [68], [69], [70]] and/or a meta-regression method – precision-effect test-precision-effect estimate with standard error (PET-PEESE) [[70], [71], [72]] – may be applied when appropriate, particularly for continuous outcomes – with a sufficient number of significant *p*-values (e.g., *p* ≥ 10) –, to explore evidential value and adjust for small-study effects [71]. This rigorous and multi-layered approach will ensure that the synthesized findings are both statistically sound and clinically meaningful.

### Certainty of evidence

The overall certainty of the evidence will be assessed using the Grading of Recommendations, Assessment, Development, and Evaluation (GRADE) approach [[73], [74], [75]]. According to GRADE, the certainty of the evidence is classified as "high", "moderate", "low", or "very low" [76]. Observational studies start as low-certainty evidence, but this rating may be downgraded or upgraded based on specific criteria [76]. The assessment will be performed using the GRADEpro Guideline Development Tool software (GRADEpro GDT, McMaster University, Canada) [77,78] to facilitate structured and transparent judgments across the five domains [30]:

• Risk of bias: downgraded by one level if 50–75 % of the included studies present a low risk of bias or by two levels if <50 % meet this criterion.
• Consistency: downgraded by one level in cases of "moderate heterogeneity" and by two levels if "high heterogeneity" is observed.
• Precision: imprecision will be assessed following the GRADE Handbook guidelines [79]. The certainty of the evidence will be downgraded by one level if the confidence interval (CI) around the effect estimate is wide and crosses a threshold of clinical decision-making, or if the total number of participants does not meet the optimal information size (OIS). If the CI includes both important benefit and harm, or if the sample size is markedly below the OIS, the certainty will be downgraded by two levels. This approach ensures closer adherence to international standards for evaluating imprecision.
• Generalizing results: downgraded by one level when the evidence is extrapolated to populations or contexts different from those originally studied.
• Publication bias: downgraded by one level when evidence of bias is detected, particularly according to Egger's regression test.

Conversely, the certainty of the evidence may be upgraded in the presence of large or very large *ES* [76]. Specifically, when the summarized *ES* – expressed as a prevalence ratio in meta-analyses of cross-sectional studies or as a relative risk in meta-analyses of cohort studies – satisfies *ES*<0.5 or *ES*>2.0, the certainty of the evidence will be upgraded by one level. If this variable satisfies *ES*<0.2 or *ES*>5.0, the certainty will be upgraded by two levels [79].

### Presentation of results

After analyzing and interpreting the results, we will submit a systematic review to a peer-reviewed scientific journal. The results will be categorized and subcategorized objectively and concisely [25,80,81], and may be presented using figures, diagrams, or other graphic elements to improve visualization [82] and enhance understanding [83]. Forest plots will display individual and pooled effect sizes (95 % CI). For meta-regression analyses, bubble plots will be used to visualize the relationship between moderators and outcomes [30]. Presenting the results in a suitable and detailed format will allow the reviewers to identify gaps in the literature and map the available evidence [84].

Interactive dashboards and complementary online materials (e.g., downloadable datasets, dynamic visualizations) will be developed and hosted on an open-access platform to enhance accessibility and user engagement, particularly for policymakers, clinicians, and researchers. To amplify the impact of the findings beyond academic audiences, a multi-pronged dissemination strategy will be adopted. This will include the development of policy briefs, executive summaries, and infographics tailored for health system stakeholders. Where applicable, the results will be presented to relevant health authorities, particularly in LMICs.

### Ethics and dissemination

Ethical approval is not typically required for systematic review studies, as they generally analyze previously published studies or publicly available evidence (i.e., secondary data). The results and conclusions obtained will be published in a peer-reviewed journal, contributing to the advancement of the research area and supporting further investigation [23].

## Strengths and limitations

As with any systematic review, this study will face certain limitations. One key challenge is the heterogeneity in how integrated care is defined and implemented across health systems and contexts, which may limit comparability and complicate the interpretation of pooled results. The quality and reporting standards of the included primary studies may vary substantially in terms of design, sample size, and completeness of outcome data. Since this review relies on secondary data, the validity of the conclusions will inherently depend on the rigor of these original studies. Moreover, publication bias – especially in LMICs, where research is often published in local or non-indexed journals – may affect the comprehensiveness of evidence capture [85]. Inconsistent terminology and incomplete reporting may also hinder the synthesis of findings across studies.

Despite these limitations, this review protocol demonstrates several significant strengths. First, it is guided by internationally recognized standards – such as PRISMA-P 2015, PRISMA-S, PRESS 2015, and GRADE – ensuring methodological transparency, reproducibility, and rigor. The study is prospectively registered in PROSPERO, and the protocol publication promotes accountability and minimizes the risk of selective reporting.

Second, the study will employ a comprehensive and validated search strategy that encompasses both primary and secondary sources, drawing from a wide range of multidisciplinary and health-specific databases. This approach is designed to maximize the retrieval of all relevant studies. Additionally, a recursive search using citation tracking of included studies will further enhance the identification of potentially relevant literature not captured by the initial search strings.

Third, the methodological design is particularly robust, incorporating subgroup and meta-regression analyses, and influence diagnostics. These techniques are designed to enhance analytical precision, mitigate potential sources of heterogeneity, and facilitate more nuanced interpretations of the data. The use of advanced statistical methods to detect and adjust for publication bias – such as funnel plots, Egger's test, Trim and Fill, *P*-curve analysis, and PET-PEESE – will enhance the reliability of the findings, particularly for continuous outcomes.

Lastly, the use of specialized software will support each phase of the systematic review process, thereby contributing to the study's efficiency, accuracy, and transparency. Together, these methodological strengths will enhance the quality and impact of the review, making it a valuable resource for informing public health strategies and integrated care models in diabetes management.

## Ethics statements

**Institutional Review Board Statement**: Not applicable.

**Informed Consent Statement**: Not applicable.

**Funding**: This study did not receive external funding.

**Data Availability Statement**: All files containing the metadata extracted from the databases (the original raw data before the deduplication process) will be publicly available as supplementary material in RIS format in the scientific repository (OSF® Platform, available at https://osf.io/). The data will only be publicly available after the manuscript containing the systematic review results has been accepted for publication. With any update of the necessary systematic review, the new complementary datasets extracted will also be made available in this same repository.

## CRediT authorship contribution statement

**Woska Pires da Costa:** Conceptualization, Methodology, Validation, Visualization, Writing – original draft, Writing – review & editing. **Heloísa de Carvalho Torres:** Conceptualization, Investigation, Methodology, Project administration, Resources, Supervision, Writing – original draft, Writing – review & editing. **Thiago Dias Sarti:** Investigation, Resources, Writing – original draft, Writing – review & editing. **Ana Paula Santana Coelho Almeida:** Investigation, Resources, Visualization, Writing – original draft, Writing – review & editing. **Sandro Rogério Rodrigues Batista:** Methodology, Resources, Validation, Writing – original draft, Writing – review & editing. **Luiz Carlos de Abreu:** Methodology, Resources, Supervision, Validation, Visualization, Writing – original draft, Writing – review & editing.

## Declaration of competing interest

The authors declare that they have no known competing financial interests or personal relationships that could have appeared to influence the work reported in this paper.

## Data Availability

No data was used for the research described in the article.

## Appendix Supplementary materials

**S1 File**: The PRISMA-P checklist for this systematic review protocol. Image, application 1

**S2 File**: Details of the Boolean search string used for each database. Image, application 2

**S3 File**: Data extraction summary form. Image, application 3
