# Supplementary material for: Effects of integrated care on health outcomes in patients with diabetes in primary healthcare: A systematic review and meta-analysis protocol
Source: MethodsX. 2025 Sep 15;15:103628. doi: 10.1016/j.mex.2025.103628 (PMC12510043; doi:10.1016/j.mex.2025.103628)
Supplement: Supplementary file 2 — S2 File: Details of the Boolean search string used for each database. [file mmc2.pdf]

Supplementary material S2. Details of Boolean search string used for each database.

Scopus™ via native interface	
**Blocks and returns**	**Search strings**
#1 (Return: ‹n_1›)	TITLE-ABS-KEY(diabetes OR diabetic OR "t2dm" OR "glucose intolerance" OR "impaired glucose tolerance" OR "non-insulin dependent diabetes" OR glycuresis OR hyperglycemia OR "high blood sugar" OR glucosuria)
#2 (Return: ‹n_2›)	TITLE-ABS-KEY("integrated care" OR "integrated healthcare" OR "integrated health-care" OR "integrated health care" OR "coordinated care" OR "coordinated healthcare" OR "coordinated health-care" OR "coordinated health care" OR "multidisciplinary care" OR "multidisciplinary healthcare" OR "multidisciplinary health-care" OR "multidisciplinary health care" OR "comprehensive care" OR "comprehensive healthcare" OR "comprehensive health-care" OR "comprehensive health care" OR "collaborative care" OR "collaborative healthcare" OR "collaborative health-care" OR "collaborative health care" OR "interprofessional care" OR "interprofessional healthcare" OR "interprofessional health-care" OR "interprofessional health care" OR "team-based care" OR "team-based healthcare" OR "team-based health-care" OR "team-based health care" OR "continuity of care" OR "continuity of healthcare" OR "continuity of health-care" OR "continuity of health care" OR "patient-centered care" OR "patient-centered healthcare" OR "patient-centered health-care" OR "patient-centered health care" OR "chronic care model" OR "chronic healthcare model" OR "chronic health-care model" OR "chronic health care model")
#3 (Return: ‹n_3›)	TITLE-ABS-KEY("primary medical care" OR "primary care" OR "primary healthcare" OR "primary health care" OR "primary health-care" OR "family healthcare" OR "family health care" OR "family health-care" OR "family health program" OR "family healthcare program" OR "family health care program" OR "family health-care program" OR "community health services" OR "community healthcare services" OR "community health-care services" OR "general practice" OR "general healthcare practice" OR "general health care practice" OR "general health-care practice" OR "general practitioner" OR "general healthcare practitioner" OR "general health care practitioner" OR "general health-care practitioner" OR "first-level care" OR "first-level healthcare" OR "first-level health care" OR "first-level health-care" OR "ambulatory care" OR "ambulatory healthcare" OR "ambulatory health-care" OR "ambulatory health care" OR "outpatient care" OR "outpatient healthcare" OR "outpatient health-care" OR "outpatient health care")
#4 (Return: ‹n_4›)	TITLE-ABS-KEY("clinical outcome" OR "health outcome" OR "glycemic control" OR "blood glucose" OR "HbA1c" OR "hemoglobin A1c" OR "treatment adherence" OR "medication adherence" OR "self-care" OR "disease management" OR "life prospect" OR "life expectation" OR "perspective on life" OR "prospect in life" OR "expectation of life" OR "expectation for life" OR "diabetes control" OR "metabolic control" OR "complication rate" OR "diabetes complications" OR "functional status" OR "treatment outcome" OR "quality of life" OR qol OR hospitalization OR mortality OR readmission)
#5 (Return: ‹n_5›)	(#1) AND (#2) AND (#3) AND (#4)
	**Search refinement**:<br>⟨⟨Not applied⟩⟩<br>**Return after refinement**:<br>‹*N*› studies in the test carried out in ‹*date*›.

Note: TITLE-ABS-KEY expression indicates that the search was conducted simultaneously in the title, abstract, and keywords fields of the documents indexed in Scopus. A pilot return test was conducted on July 17, 2025, to assess the feasibility of the systematic review, resulting in 1,726 studies retrieved from the Scopus database.

Web of Science™ Core Collection via native interface

Blocks and returns	Search strings
#1 (Return: ‹n_1›)	TS=(diabetes OR diabetic OR "t2dm" OR "glucose intolerance" OR "impaired glucose tolerance" OR "non-insulin dependent diabetes" OR glycuresis OR hyperglycemia OR "high blood sugar" OR glucosuria)
#2 (Return: ‹n_2›)	TS=("integrated care" OR "integrated healthcare" OR "integrated health-care" OR "integrated health care" OR "coordinated care" OR "coordinated healthcare" OR "coordinated health-care" OR "coordinated health care" OR "multidisciplinary care" OR "multidisciplinary healthcare" OR "multidisciplinary health-care" OR "multidisciplinary health care" OR "comprehensive care" OR "comprehensive healthcare" OR "comprehensive health-care" OR "comprehensive health care" OR "collaborative care" OR "collaborative healthcare" OR "collaborative health-care" OR "collaborative health care" OR "interprofessional care" OR "interprofessional healthcare" OR "interprofessional health-care" OR "interprofessional health care" OR "team-based care" OR "team-based healthcare" OR "team-based health-care" OR "team-based health care" OR "continuity of care" OR "continuity of healthcare" OR "continuity of health-care" OR "continuity of health care" OR "patient-centered care" OR "patient-centered healthcare" OR "patient-centered health-care" OR "patient-centered health care" OR "chronic care model" OR "chronic healthcare model" OR "chronic health-care model" OR "chronic health care model")
#3 (Return: ‹n_3›)	TS=("primary medical care" OR "primary care" OR "primary healthcare" OR "primary health care" OR "primary health-care" OR "family healthcare" OR "family health care" OR "family health-care" OR "family health program" OR "family healthcare program" OR "family health care program" OR "family health-care program" OR "community health services" OR "community healthcare services" OR "community health-care services" OR "general practice" OR "general healthcare practice" OR "general health care practice" OR "general health-care practice" OR "general practitioner" OR "general healthcare practitioner" OR "general health care practitioner" OR "general health-care practitioner" OR "first-level care" OR "first-level healthcare" OR "first-level health care" OR "first-level health-care" OR "ambulatory care" OR "ambulatory healthcare" OR "ambulatory health-care" OR "ambulatory health care" OR "outpatient care" OR "outpatient healthcare" OR "outpatient health-care" OR "outpatient health care")
#4 (Return: ‹n_4›)	TS=("clinical outcome" OR "health outcome" OR "glycemic control" OR "blood glucose" OR "HbA1c" OR "hemoglobin A1c" OR "treatment adherence" OR "medication adherence" OR "self-care" OR "disease management" OR "life prospect" OR "life expectation" OR "perspective on life" OR "prospect in life" OR "expectation of life" OR "expectation for life" OR "diabetes control" OR "metabolic control" OR "complication rate" OR "diabetes complications" OR "functional status" OR "treatment outcome" OR "quality of life" OR qol OR hospitalization OR mortality OR readmission)
#5 (Return: ‹n_5›)	(#1) AND (#2) AND (#3) AND (#4)

Search refinement:

⟨⟨Not applied⟩⟩

Return after refinement:

‹*N*› studies in the test carried out in ‹*date*›.

Note: TS indicates a search in the Topic field of Web of Science, covering the title, abstract, and keywords. A pilot return test was conducted on July 17, 2025, to assess the feasibility of the systematic review, resulting in 857 studies retrieved from the Web of Science database.

MEDLINE/PubMed® via interface of the National Library of Medicine® (NLM®)	
**Blocks and returns**	**Search strings**
#1 (Return: ‹n_1›)	("Diabetes Mellitus"[MeSH] OR diabetes[tiab] OR diabetic[tiab] OR "Diabetes Mellitus, Type 2"[MeSH] OR "t2dm"[tiab] OR "Glucose Intolerance"[MeSH] OR "glucose intolerance"[tiab] OR "impaired glucose tolerance"[tiab] OR "non-insulin dependent diabetes"[tiab] OR glycuresis[tiab] OR "Hyperglycemia"[MeSH] OR hyperglycemia[tiab] OR "high blood sugar"[tiab] OR "Glucosuria"[MeSH] OR glucosuria[tiab])
#2 (Return: ‹n_2›)	("integrated care"[tiab] OR "integrated healthcare"[tiab] OR "integrated health-care"[tiab] OR "integrated health care"[tiab] OR "coordinated care"[tiab] OR "coordinated healthcare"[tiab] OR "coordinated health-care"[tiab] OR "coordinated health care"[tiab] OR "Delivery of Health Care, Integrated"[MeSH] OR "multidisciplinary care"[tiab] OR "multidisciplinary healthcare"[tiab] OR "multidisciplinary health-care"[tiab] OR "multidisciplinary health care"[tiab] OR "comprehensive care"[tiab] OR "comprehensive healthcare"[tiab] OR "comprehensive health-care"[tiab] OR "comprehensive health care"[tiab] OR "collaborative care"[tiab] OR "collaborative healthcare"[tiab] OR "collaborative health-care"[tiab] OR "collaborative health care"[tiab] OR "interprofessional care"[tiab] OR "interprofessional healthcare"[tiab] OR "interprofessional health-care"[tiab] OR "interprofessional health care"[tiab] OR "team-based care"[tiab] OR "team-based healthcare"[tiab] OR "team-based health-care"[tiab] OR "team-based health care"[tiab] OR "continuity of care"[tiab] OR "continuity of healthcare"[tiab] OR "continuity of health-care"[tiab] OR "continuity of health care"[tiab] OR "Continuity of Patient Care"[MeSH] OR "Patient-Centered Care"[MeSH] OR "patient-centered care"[tiab] OR "patient-centered healthcare"[tiab] OR "patient-centered health-care"[tiab] OR "patient-centered health care"[tiab] OR "chronic care model"[tiab] OR "chronic healthcare model"[tiab] OR "chronic health-care model"[tiab] OR "chronic health care model"[tiab])
#3 (Return: ‹n_3›)	("primary medical care"[tiab] OR "primary care"[tiab] OR "primary healthcare"[tiab] OR "Primary Health Care"[MeSH] OR "primary health care"[tiab] OR "primary health-care"[tiab] OR "family healthcare"[tiab] OR "family health care"[tiab] OR "family health-care"[tiab] OR "family health program"[tiab] OR "family healthcare program"[tiab] OR "family health care program"[tiab] OR "family health-care program"[tiab] OR "Community Health Services"[MeSH] OR "community health services"[tiab] OR "community healthcare services"[tiab] OR "community health-care services"[tiab] OR "General Practice"[MeSH] OR "general practice"[tiab] OR "general healthcare practice"[tiab] OR "general health care practice"[tiab] OR "general health-care practice"[tiab] OR "general practitioner"[tiab] OR "general healthcare practitioner"[tiab] OR "general health care practitioner"[tiab] OR "general health-care practitioner"[tiab] OR "first-level care"[tiab] OR "first-level healthcare"[tiab] OR "first-level health care"[tiab] OR "first-level health-care"[tiab] OR "Ambulatory Care"[MeSH] OR "ambulatory care"[tiab] OR "ambulatory healthcare"[tiab] OR "ambulatory health-care"[tiab] OR "ambulatory health care"[tiab] OR "outpatient care"[tiab] OR "outpatient healthcare"[tiab] OR "outpatient health-care"[tiab] OR "outpatient health care"[tiab])
#4 (Return: ‹n_4›)	("clinical outcome"[tiab] OR "health outcome"[tiab] OR "glycemic control"[tiab] OR "blood glucose"[tiab] OR "HbA1c"[tiab] OR "hemoglobin A1c"[tiab] OR "treatment adherence"[tiab] OR "Medication Adherence"[MeSH] OR "medication adherence"[tiab] OR "Self Care"[MeSH] OR "self-care"[tiab] OR "Disease Management"[MeSH] OR "disease management"[tiab] OR "life prospect"[tiab] OR "life expectation"[tiab] OR "perspective on life"[tiab] OR "prospect in life"[tiab] OR "expectation of life"[tiab] OR "expectation for life"[tiab] OR "diabetes control"[tiab] OR "metabolic control"[tiab] OR "complication rate"[tiab] OR "Diabetes Complications"[MeSH] OR "Comorbidity"[MeSH] OR "diabetes complications"[tiab] OR "Activities of Daily Living"[MeSH] OR "functional status"[tiab] OR "Treatment Outcome"[MeSH] OR "treatment outcome"[tiab] OR "Quality of Life"[MeSH] OR "quality of life"[tiab] OR qol[tiab] OR "Hospitalization"[MeSH] OR hospitalization[tiab] OR "Mortality"[MeSH] OR mortality[tiab] OR "Patient Readmission"[MeSH] OR readmission[tiab])
#5 (Return: ‹n_5›)	(#1) AND (#2) AND (#3) AND (#4)
	**Search refinement**:<br>⟨⟨Not applied⟩⟩<br>**Return after refinement**:<br>‹*N*› studies in the test carried out in ‹*date*›.

Note: MeSH is the acronym for Medical Subject Headings; [tiab] is the shorthand for [Title/Abstract] in PubMed searches. A pilot return test was conducted on July 17, 2025, to assess the feasibility of the systematic review, resulting in 3,756 studies retrieved from the PubMed.

Embase™ via native interface	
**Blocks and returns**	**Search strings**
#1 (Return: ‹n_1›)	('diabetes mellitus'/exp OR 'diabetes':ab,ti,kw OR 'diabetic':ab,ti,kw OR 't2dm':ab,ti,kw OR 'glucose intolerance':ab,ti,kw OR 'impaired glucose tolerance':ab,ti,kw OR 'glycuresis':ab,ti,kw OR 'hyperglycemia':ab,ti,kw OR 'high blood sugar':ab,ti,kw OR 'glucosuria')
#2 (Return: ‹n_2›)	('integrated care'/exp OR 'integrated care':ab,ti,kw OR 'integrated healthcare':ab,ti,kw OR 'integrated health-care':ab,ti,kw OR 'integrated health care':ab,ti,kw OR 'coordinated care':ab,ti,kw OR 'coordinated healthcare':ab,ti,kw OR 'coordinated health-care':ab,ti,kw OR 'coordinated health care':ab,ti,kw OR 'multidisciplinary care':ab,ti,kw OR 'multidisciplinary healthcare':ab,ti,kw OR 'multidisciplinary health-care':ab,ti,kw OR 'multidisciplinary health care':ab,ti,kw OR 'comprehensive care':ab,ti,kw OR 'comprehensive healthcare':ab,ti,kw OR 'comprehensive health-care':ab,ti,kw OR 'comprehensive health care':ab,ti,kw OR 'collaborative care':ab,ti,kw OR 'collaborative healthcare':ab,ti,kw OR 'collaborative health-care':ab,ti,kw OR 'collaborative health care':ab,ti,kw OR 'interprofessional care':ab,ti,kw OR 'interprofessional healthcare':ab,ti,kw OR 'interprofessional health-care':ab,ti,kw OR 'interprofessional health care':ab,ti,kw OR 'team-based care':ab,ti,kw OR 'team-based healthcare':ab,ti,kw OR 'team-based health-care':ab,ti,kw OR 'team-based health care':ab,ti,kw OR 'continuity of care':ab,ti,kw OR 'continuity of healthcare':ab,ti,kw OR 'continuity of health-care':ab,ti,kw OR 'continuity of health care':ab,ti,kw OR 'patient-centered care':ab,ti,kw OR 'patient-centered healthcare':ab,ti,kw OR 'patient-centered health-care':ab,ti,kw OR 'patient-centered health care':ab,ti,kw OR 'chronic care model':ab,ti,kw OR 'chronic healthcare model':ab,ti,kw OR 'chronic health-care model':ab,ti,kw OR 'chronic health care model')
#3 (Return: ‹n_3›)	('primary medical care':ab,ti,kw OR 'primary care':ab,ti,kw OR 'primary healthcare':ab,ti,kw OR 'primary health care'/exp OR 'primary health care':ab,ti,kw OR 'primary health-care':ab,ti,kw OR 'family healthcare':ab,ti,kw OR 'family health care':ab,ti,kw OR 'family health-care':ab,ti,kw OR 'family health program':ab,ti,kw OR 'family healthcare program':ab,ti,kw OR 'family health care program':ab,ti,kw OR 'family health-care program':ab,ti,kw OR 'general practice'/exp OR 'community health services':ab,ti,kw OR 'community healthcare services':ab,ti,kw OR 'community health-care services':ab,ti,kw OR 'general practice'/exp OR 'general practice':ab,ti,kw OR 'general healthcare practice':ab,ti,kw OR 'general health care practice':ab,ti,kw OR 'general health-care practice':ab,ti,kw OR 'general practitioner':ab,ti,kw OR 'general healthcare practitioner':ab,ti,kw OR 'general health care practitioner':ab,ti,kw OR 'general health-care practitioner':ab,ti,kw OR 'first-level care':ab,ti,kw OR 'first-level healthcare':ab,ti,kw OR 'first-level health care':ab,ti,kw OR 'first-level health-care':ab,ti,kw OR 'ambulatory care':ab,ti,kw OR 'ambulatory healthcare':ab,ti,kw OR 'ambulatory health-care':ab,ti,kw OR 'ambulatory health care':ab,ti,kw OR 'outpatient care':ab,ti,kw OR 'outpatient healthcare':ab,ti,kw OR 'outpatient health-care':ab,ti,kw OR 'outpatient health care')
#4 (Return: ‹n_4›)	('clinical outcome':ab,ti,kw OR 'health outcome':ab,ti,kw OR 'glycemic control':ab,ti,kw OR 'blood glucose control'/exp OR 'blood glucose':ab,ti,kw OR 'HbA1c':ab,ti,kw OR 'hemoglobin A1c':ab,ti,kw OR 'treatment adherence':ab,ti,kw OR 'medication adherence':ab,ti,kw OR 'self-care':ab,ti,kw OR 'disease management':ab,ti,kw OR 'life prospect':ab,ti,kw OR 'life expectation':ab,ti,kw OR 'perspective on life':ab,ti,kw OR 'prospect in life':ab,ti,kw OR 'expectation of life':ab,ti,kw OR 'expectation for life':ab,ti,kw OR 'diabetes control':ab,ti,kw OR 'metabolic control':ab,ti,kw OR 'complication rate':ab,ti,kw OR 'diabetes complications':ab,ti,kw OR 'functional status':ab,ti,kw OR 'treatment outcome'/exp OR 'treatment outcome':ab,ti,kw OR 'quality of life'/exp OR 'quality of life':ab,ti,kw OR 'qol':ab,ti,kw OR 'hospitalization'/exp OR 'hospitalization':ab,ti,kw OR 'mortality'/exp OR 'mortality':ab,ti,kw OR 'readmission'/exp OR 'readmission':ab,ti,kw)
#5 (Return: ‹n_5›)	(#1) AND (#2) AND (#3) AND (#4)
	**Search refinement**: ⟨⟨Not applied⟩⟩ **Return after refinement**: ‹*N*› studies in the test carried out in ‹*date*›.

Note: The use of 'ab,ti,kw' indicates that the search considered the title, abstract, and keyword fields in Embase; the use of '/exp' indicates that the term is exploded within the hierarchical tree of the controlled vocabulary Emtree, meaning that all more specific terms related to that concept are also automatically included in the search. A pilot return test was conducted on July 17, 2025, to assess the feasibility of the systematic review, resulting in 1,276 studies retrieved from the Embase database.

Cumulative Index to Nursing and Allied Health Literature (CINAHL®) via EBSCOhost interface	
**Blocks and returns**	**Search strings**
#1 (Return: ‹n_1›)	XB (diabetes OR diabetic OR "t2dm" OR "glucose intolerance" OR "impaired glucose tolerance" OR "non-insulin dependent diabetes" OR glycuresis OR hyperglycemia OR "high blood sugar" OR glucosuria)
#2 (Return: ‹n_2›)	XB ("integrated care" OR "integrated healthcare" OR "integrated health-care" OR "integrated health care" OR "coordinated care" OR "coordinated healthcare" OR "coordinated health-care" OR "coordinated health care" OR "multidisciplinary care" OR "multidisciplinary healthcare" OR "multidisciplinary health-care" OR "multidisciplinary health care" OR "comprehensive care" OR "comprehensive healthcare" OR "comprehensive health-care" OR "comprehensive health care" OR "collaborative care" OR "collaborative healthcare" OR "collaborative health-care" OR "collaborative health care" OR "interprofessional care" OR "interprofessional healthcare" OR "interprofessional health-care" OR "interprofessional health care" OR "team-based care" OR "team-based healthcare" OR "team-based health-care" OR "team-based health care" OR "continuity of care" OR "continuity of healthcare" OR "continuity of health-care" OR "continuity of health care" OR "patient-centered care" OR "patient-centered healthcare" OR "patient-centered health-care" OR "patient-centered health care" OR "chronic care model" OR "chronic healthcare model" OR "chronic health-care model" OR "chronic health care model")
#3 (Return: ‹n_3›)	XB ("primary medical care" OR "primary care" OR "primary healthcare" OR "primary health care" OR "primary health-care" OR "family healthcare" OR "family health care" OR "family health-care" OR "family health program" OR "family healthcare program" OR "family health care program" OR "family health-care program" OR "community health services" OR "community healthcare services" OR "community health-care services" OR "general practice" OR "general healthcare practice" OR "general health care practice" OR "general health-care practice" OR "general practitioner" OR "general healthcare practitioner" OR "general health care practitioner" OR "general health-care practitioner" OR "first-level care" OR "first-level healthcare" OR "first-level health care" OR "first-level health-care" OR "ambulatory care" OR "ambulatory healthcare" OR "ambulatory health-care" OR "ambulatory health care" OR "outpatient care" OR "outpatient healthcare" OR "outpatient health-care" OR "outpatient health care")
#4 (Return: ‹n_4›)	XB ("clinical outcome" OR "health outcome" OR "glycemic control" OR "blood glucose" OR "HbA1c" OR "hemoglobin A1c" OR "treatment adherence" OR "medication adherence" OR "self-care" OR "disease management" OR "life prospect" OR "life expectation" OR "perspective on life" OR "prospect in life" OR "expectation of life" OR "expectation for life" OR "diabetes control" OR "metabolic control" OR "complication rate" OR "diabetes complications" OR "functional status" OR "treatment outcome" OR "quality of life" OR qol OR hospitalization OR mortality OR readmission)
#5 (Return: ‹n_5›)	(#1) AND (#2) AND (#3) AND (#4)
	**Search refinement**:<br>⟨⟨Not applied⟩⟩<br>**Return after refinement**:<br>‹*N*› studies in the test carried out in ‹*date*›.

Note: XB indicates that the search was conducted in the Title and Abstract fields of CINAHL via EBSCOhost. A pilot return test was conducted on July 17, 2025, to assess the feasibility of the systematic review, resulting in 300 studies retrieved from the CINAHL database.
