# Supplementary material for: Effects of integrated care on health outcomes in patients with diabetes in primary healthcare: A systematic review and meta-analysis protocol
Source: MethodsX. 2025 Sep 15;15:103628. doi: 10.1016/j.mex.2025.103628 (PMC12510043; doi:10.1016/j.mex.2025.103628)
Supplement: Supplementary file 3 — S3 File: Data extraction summary form. [file mmc3.docx]

**Supplementary material S3.** Data extraction summary form of the studies included in this systematic review.

| **(A)** | **(B)** | **(C)** | **(D)** | **(E)** | **(F)** | **(G)** | **(H)** | **(I)** | **(J)** | **(K)** | **(L)** | **(M)** | **(N)** | **(O)** |
| --- | --- | --- | --- | --- | --- | --- | --- | --- | --- | --- | --- | --- | --- | --- |
| **Citation**  **detail** | **Study**  **location** | **Study**  **objective** | **Population** | **Sample size**  **and**  **time frame** | **Description**  **of the**  **integrated care** | **Primary**  **healthcare**  **context** | **Study design**  **and**  **methods** | **Primary outcome** | **Secondary**  **outcomes** | **Main**  **results** | **Stratification**  **and disparities**  **identified** | **Reported**  **gaps** | **Limitations** | **Implications**  **and/or**  **recommendations** |
|  |  |  |  |  |  |  |  |  |  |  |  |  |  |  |
| John & Lee (2022) ^*^ | Greece | To analyze the impact of integrated care programs on glycemic control and hospital admissions in diabetic patients. | Adults with type 2 diabetes. | *n* = 875  2015–2017 | Multidisciplinary team, individualized care plans, shared electronic records. | Urban primary care centers with chronic care management structures. | Quasi-experimental, matched controls, retrospective data. | Glycemic control (HbA1c):  Mean decreased from 8.7% at baseline to 7.2% after 12 months, corresponding to a –1.5% mean reduction  (*p* < 0.001). | a) Treatment adherence improved from 62% at baseline to 81% at the latest follow-up, representing a  19-percentage point  increase  (OR=2.35; 95% CI: 1.80–3.07).  b) The annual hospitalization rate decreased from 18.4% to 10.6%, corresponding to a 42% relative reduction  (RR=0.58; 95% CI: 0.44–0.77). | Improved glycemic control and reduced hospital use. | The benefits are stronger in patients with low income and comorbidities. | Need for long-term evaluation and standard definitions. | Small sample from one region, short follow-up. | The study suggests expansion of integrated care to underserved areas. |
|  |  |  |  |  |  |  |  |  |  |  |  |  |  |  |

**Note:** * Hypothetical example data.
